# Dietary intake of vitamin D during adolescence and risk of adult onset systemic lupus erythematosus and rheumatoid arthritis

**DOI:** 10.1186/1546-0096-10-S1-A30

**Published:** 2012-07-13

**Authors:** Linda T Hiraki, Karen H Costenbader, Kassandra L Munger, Elizabeth W Karlson

**Affiliations:** 1 Brigham and Women's Hospital, Harvard Medical School, Boston, MA, USA; 2 Harvard School of Public Health, Boston, MA, USA

## Purpose

Early life exposures have been implicated in the etiology of rheumatoid arthritis (RA) and systemic lupus erythematosus (SLE). Vitamin D has immunomodulatory effects with implications as an etiologic and therapeutic factor in several autoimmune diseases including SLE and RA. There are no studies on vitamin D intake during adolescence and the risk of adult onset SLE and RA.

## Purpose

We examined the relationship between reported vitamin D intake during adolescence and incidence of adult onset RA and SLE in two prospective cohort studies, the Nurses’ Health Study (NHS) and Nurses’ Health Study II (NHSII).

## Methods

Previously validated food frequency questionnaires (FFQ) on high school diet completed by 75,458 NHS participants in 1986 and 45,948 NHSII participants in 1998 (maximum recall of 47 and 35 years respectively), were used to calculate nutrient intakes during adolescence. Incident RA and SLE cases were confirmed medical record review. Cox-proportional hazards regression relative risks (RR) and 95% CI were calculated for quintiles of adolescent dietary vitamin D intake and incident RA, incident SLE. Calorie adjusted and multivariate adjusted analyses were completed, with adjustment for demographic, dietary and sun-exposure related factors. Random effects models were used to obtain a single combined estimate of association across the two cohorts.

## Results

Incident RA was confirmed in 652 NHS and 126 NHSII participants (total 778), and incident SLE was confirmed in 122 NHS and 52 NHSII participants (total 174) over a mean followup of 329 and 207 months for NHS and NHSII respectively. Calorie-adjusted and multivariate-adjusted models did not show significant associations between adolescent vitamin D intake and risk of adult onset RA and SLE when comparing the 5th quintile of vitamin D intake to the 1st quintile (Table 1).

**Table 1:** Estimated hazard ratios and 95% CI of associated between adolescent vitamin D intake and incident RA and SLE

	NHS	NHSII	Pooled
Number of RA cases	652	126	778
HR (95% CI) Energy adjusted Total Vitamin D	1.03 (0.8, 1.32)	1.06 (0.62, 1.82)	1.03 (0.82, 1.3)
HR (95% CI) Multivariate adjusted Total Vitamin D	0.87 (0.6, 1.26)	0.96 (0.51, 1.81)	0.87 (0.51, 1.81)
Number of SLE cases	122	522	174
HR (95% CI) Energy adjusted Total Vitamin D	1.09 (0.62, 1.95)	1.08 (0.47, 2.45)	1.03 (0.65, 1.63)
HR (95% CI) Multivariate adjusted Total Vitamin D	1.16 (0.52, 2.56)	0.69 (0.25, 1.88)	0.87 (0.5, 1.46)

## Conclusion

We did not find associations between reported dietary intake of vitamin D during adolescence and risk of RA or SLE in adulthood. This may be due to inaccurate reporting of adolescent diet, focus on a short time period of exposure and unavailable serum vitamin D levels during this exposure period of interest. Small numbers of incident cases also reduced our power to detect an association if one existed.

## Disclosure

Linda T. Hiraki: None; Karen H. Costenbader: None; Kassandra L. Munger: None; Elizabeth W. Karlson: None.

